# Impact of the Physiological Parameters of the Oral Cavity on the Mechanical Properties of Zinc-stabilized and Zinc-free Adhesive Creams: A Comparative In Vitro Study

**DOI:** 10.3290/j.jad.c_1871

**Published:** 2025-03-10

**Authors:** Josephine Reymann, Anantha Narayanan Ramakrishnan, Christopher Ludtka, Jeremias Hey, Andreas Kiesow, Stefan Schwan

**Affiliations:** a Josephine Reymann PhD student, Department of Operative Dentistry and Periodontology, Martin Luther University Halle-Wittenberg, Halle, Germany. Research idea, experimental design, performed the experiments, wrote the manuscript, contributed substantially to discussion and Researcher, Fraunhofer Institute for Microstructure of Materials and Systems IMWS, Department of Biological and Macromolecular Materials, Halle, Germany; b Anantha Narayanan Ramakrishnan Researcher, Hochschule Merseburg, University of Applied Sciences, Department of Engineering and Natural Sciences, Merseburg, Germany; and PhD student, University of Stuttgart, Institute for Modelling and Simulation of Biomechanical Systems, Faculty of Civil and Environmental Engineering, Stuttgart, Germany. Research idea, hypothesis, visualization of results, wrote the manuscript, proofread the manuscript, contributed substantially to discussion.; c Christopher Ludtka Researcher, University of Florida, J. Crayton Pruitt Family Department of Biomedical Engineering, Gainesville, USA. Proofread the manuscript, formal analysis, visualization of results. Supervision, validation of results, formal analysis.; d Jeremias Hey Professor, Department of Prosthetic Dentistry, University School of Dental Medicine, Martin Luther University Halle-Wittenberg, Halle, Germany. Project administration, formal analysis and proofread the manuscript.; e Andreas Kiesow Researcher, Fraunhofer Institute for Microstructure of Materials and Systems IMWS, Department of Biological and Macromolecular Materials, Halle, Germany. Project administration, formal analysis and proofread the manuscript.; f Stefan Schwan Researcher, Fraunhofer Institute for Microstructure of Materials and Systems IMWS, Department of Biological and Macromolecular Materials, Halle, Germany and Professor, Hochschule Merseburg, University of Applied Sciences, Department of Engineering and Natural Sciences, Merseburg, Germany. Research idea, experimental design, supervision, validation of results, co-wrote and proofread the manuscript, contributed substantially to discussion.; g Swelling (%)

**Keywords:** bite force, damping, denture retention, physiological parameters, zinc-free denture adhesive

## Abstract

**Purpose:**

The current trend in denture adhesives is shifting toward zinc-free formulations due to the significant health concerns associated with zinc. Studies have focused on the retention of these zinc-free denture adhesives; however, there is a dearth of literature regarding their damping performance. This study analyzes the impact of oral cavity physiological parameters: temperature, pH, and swelling ratio on the mechanical properties of zinc-stabilized and zinc-free denture adhesives and examines the role zinc plays in influencing the adhesive behavior. This study investigates how underlying mechanical properties of adhesive cream change for zinc-stabilized and zinc-free denture adhesives. The relative damping and its variation from exposure to physiological conditions in the oral cavity can significantly assist in the design of dentures to reduce the discomfort experienced by denture wearers.

**Materials and Methods:**

The relative damping of the zinc-stabilized and zinc-free denture adhesives was investigated with the loss modulus values, which were evaluated using rheological frequency sweep tests. The tests were performed by maintaining the denture adhesives at specified values of temperature, pH, and swelling ratio, and over a frequency range of 0.01 Hz to 10 Hz, which included the average frequencies of human chewing / bite forces reported in existing literature.

**Results:**

Zinc-stabilized denture adhesive showed a larger fluctuation of loss modulus values compared to the zinc-free formulation with respect to pH, temperature, and swelling ratios. The zinc-free denture adhesive showed higher damping behavior at frequencies below 0.7 Hz, whereas the zinc-stabilized denture adhesive showed higher damping behavior above loading frequencies of 0.7–1 Hz. Both the adhesives showed varying behavior on either side of the bite force spectrum in terms of relative damping of the applied bite.

**Conclusions:**

The damping or cushioning effect provided by denture adhesives can reduce pain experienced and assist dental practitioners in better supporting denture wearers.

Tooth loss and edentulism especially are conditions that have become less common in recent years but have not completely disappeared.^14,22^ Tooth loss can have various causes and is mostly associated with caries or periodontitis.^34^ Studies have also shown that the following risk factors can be associated with edentulism: gender, age, education level, social status, and even general diseases such as diabetes or hypertension.^34^ The care for gap dentition or edentulous patients classically includes the use of partial or complete dentures. Partial dentures are anchored to the remaining teeth, eg, by means of clasps, whereas complete dentures provide a firm fit via a denture base that is congruent with the tegument, muscular adaptation, and occlusal support.^8^ Several studies have shown that the retention and stability of these removable prostheses can be improved by the use of adhesive cream.^2,9,15,28,30,33,38^ The term “adhesive cream” has been previously defined by Munoz et al^28^ as a “mixture of short- and long-chain synthetic polymers that hydrate and increase in volume to fill the voids between the denture and the mucosal tissues”. A distinction between denture adhesives is made between the soluble products such as powders or creams, and the insoluble products such as pads.^30^ The most commonly used types are the powders creams, followed by pads.^5^ These differ both in their composition as well as in their handling. The soluble products primarily consist of sodium carboxymethylcellulose or other synthetic polymers,^30^ which swell in combination with water, increasing in volume.^2^ In addition, ingredients such as mineral oil, petroleum, and polyethylene oxide are often present as fillers,^30^ and also act as binding materials.^13,16^

Vegetable gums, like Karaya gum, were initially used in the development of denture adhesives. Over subsequent decades synthetic denture adhesives consisting of divalent cations like zinc salts have been introduced to enhance adhesive behavior.^17^ However, researchers have reported that excessive ingestion of zinc contributes to neurologic disease, and as such, the focus has shifted toward zinc-free denture adhesives.^29,49^ Another potential adverse effect of zinc is that it can lead to a clinical deficiency of the trace element copper and consequently result in pathologies such as myelopathies.^3,10,36^ In literature, various studies have discussed the retention behavior of dentures using denture adhesives;^31,40,41,42,48^ however, the effect of zinc versus zinc-free denture adhesives on the retention of mandibular dentures has remained relatively unexplored.

Denture adhesive cream is applied between the prosthesis and the tegument, supporting the general retention mechanisms of the prosthesis and helping to dampen the transmitted forces on the bone by acting like a cushion.^30^ This damping of the transmitted forces has been shown to reduce the functional stress levels on the oral mucosa and consequently also on the underlying bone and hence is argued to reduce pain.^1,6,45,46,50^ The damping behavior is based on the ability of the material to dissipate the elastic strain energy due to the applied bite and chewing forces.^52^ The viscoelastic properties of denture adhesive formulation play a significant role in the damping of these forces. The cream not only increases the stability and retention of the prosthesis but also promotes patient self-confidence.30 Patients feel more confident in social interactions because they do not have to fear their prosthesis shifting or falling out while talking or eating.^2,21,25,33,38^ Ideally, an adhesive cream should work for 12 to 16 hours to cover the active period of a day.^2^ Therefore, the cream is exposed to the physiological conditions of the oral cavity for numerous hours each day. These physiological conditions are significantly impacted by both the presence of saliva, which as a liquid has a certain electrolyte content and therefore pH value, as well as the prevailing temperature within the oral cavity. It has been found that saliva produced by the salivary glands has an average pH value of 6.8.^26^ This value can change temporarily to either a more acidic or alkaline environment due to the consumption of various foods or beverages or by breathing through the nose versus through the mouth.^26^ The flow rate of saliva can also lead to changes in pH.^7,26^ The temperature prevailing in the oral cavity is also influenced by the causes just noted (ie, food, beverage, respiratory pathway) and can vary from 1.62°C to 65.43°C.^4,27^ On average, however, a temperature of approximately 34°C has been reported for the oral cavity in various studies.^7^ The variation of temperature, pH, and swelling ratio have also been incorporated into basic mathematical models predicting the influence on the mechanical properties, including the damping behavior of denture adhesives in literature.^43,44^ These models could potentially be used to evaluate the impact of the damping of the adhesive formulations for various dynamic bite and chewing scenarios.

In this study, two soluble adhesive creams were compared: one that was zinc-stabilized and another that was zinc-free. The aim was to identify the changes in the mechanical properties of these adhesive creams due to the physiological conditions within the oral cavity (temperature, pH, and swelling due to saliva), and to examine the role that zinc plays in influencing the behavior of these two different formulations. Furthermore, the objective of this work was to compare the relative damping behavior of the two adhesives tested under the influence of the aforementioned physiological factors.

## MATERIALS AND METHODS

In this study two commercially available adhesive creams (one with zinc and the other zinc-free) were evaluated using rheological tests to determine their storage and loss modulus values, which were then used to understand their mechanical damping behavior in response to an applied bite force. The two selected creams were evaluated using a parallel-plate rheometer to assess their response under the influence of relevant physiological parameters of the oral cavity. Specifically, these were: the temperature during the rheological measurements; the pH of the artificial saliva; and the degree of swelling after immersion in artificial saliva. The experimental matrix for the three physiological parameters taken into consideration in this study are based on the work of Koehler et al.^23^ This study further expands this work and compares the zinc-stabilized and zinc-free formulations when exposed to the three physiological parameters. Initially a null hypothesis that there is no significant difference between the zinc-stabilized and the zinc-free formulation due to the variation in physiological predictor variables of temperature, pH, and swelling ratio is assumed, and this is evaluated based on the mechanical response in terms of the loss modulus values of the two formulations.

### Denture Adhesive

In this study, two representative formulations of denture creams were investigated. Zinc creams were represented by Blend-a-dent Super Adhesive Cream Neutral, hereinafter referred to as the zinc-stabilized formulation, while Super Poligrip® Ultra Fresh Denture Adhesive Cream served as the zinc-free formulation. Ingredients of the zinc-stabilized formulation include calcium/zinc PVM/MA copolymer, cellulose gum, paraffin oil, petrolatum, and silica gel. Meanwhile, the zinc-free product contained calcium/sodium PVM/MA copolymer, cellulose gum, petrolatum, mineral oil, aroma, Red 30 lake, and Red 7 lake.

### Measuring the Influence of pH, Temperature, and Saliva

The adhesive specimens were tested for the influence of an acidic or alkaline medium by maintaining them at pH 2 and pH 10, respectively. The adhesives were also evaluated under neutral pH conditions at pH 7. To achieve this, the adhesive specimens were kept immersed in an acidic, alkaline, or neutral pH solution before the rheological testing. Artificial saliva was prepared for the experiments, following the formulation of Pratten et al.^37^ For this purpose, 5 g/L proteose peptone, 2.5 g/L porcine gastric mucus, 1 g/L “Lab-lemco” powder, 2 g/L yeast extract, 3.5 g/L sodium chloride, 0.2 g/L calcium chloride, and 0.2 g/L potassium chloride were dissolved in 1 L of distilled water and autoclaved at 121°C for 15 min. Then, the artificial saliva was completed by adding 1.25 ml of 40% urea. As saliva with different pH values was needed for measurements, pH was adjusted with 20% HCl solution and 10% NaOH solution while using a pH meter (accuracy to one-hundredth). Thus, artificial saliva was prepared at the noted pH values (pH 2, 7, and 10).

Before the rheological measurements could be made, the swelling rates of the selected creams had to be determined. For this purpose, the mass increase of the adhesive creams was determined after they had been soaked in artificial saliva. The measurements were carried out over a period of 2.5 h and at room temperature (23°C). For this purpose, approximately 1.6 mg of the adhesive cream was added to each individual cell sieve and then placed in the artificial saliva. The mass was determined using a precision balance every 10 minutes. For each product and pH, the mass increase was determined a total of six times. A software program (OriginPro 2019; OriginLab Corporation) was used to plot these values and fit a suitable function curve to the weight measurements. This data was then used to estimate the time that an adhesive cream sample must be immersed in saliva to reach a certain swelling percentage. In order to simulate the combined influence of the physiological conditions of the oral cavity on the cream products, the temperature at which the rheological measurements took place was also adjusted in addition to the pH of the saliva. For each pH value and swelling percentage, measurements were made in the range from 17°C to 52°C, with the temperature increasing in increments of 5°C. Within a range of 17°C to 52°C, one measurement was made for each pH value and swelling percentage.

### Rheological Measurements

The rotary rheometer used was a Thermo Scientific HAAKE model, comprised of two congruent circular parallel plates with a diameter of 35 mm. During measurements, the plates are separated from one another by a distance of 1 mm. The frequency range was set at 0.01 Hz to 10 Hz and the shear rate at 0.3%. The desired measurement temperature was set before each individual measurement. When the temperature was changed, care was taken to wait at least 10 min before starting the actual measurement so as to ensure that the plates had reached the desired temperature. Additionally, the zero point (ie, the point of contact between the two plates) was recalibrated after each temperature change. In this way, the thermal expansion of the metal plates was taken into account and a measurement gap of 1 mm could be guaranteed for each sample assessment. To prepare the adhesive cream samples for measurement, they were immersed in artificial saliva. The immersion time in the artificial saliva was according to the data and fit curve obtained in the swelling test. For this, the prepared cell sieves, a six-well plate, and the artificial saliva (pH 2, 7, or 10) were used, and the mass increase of the cream confirmed prior to testing. The sample was removed from the saliva after the time for the target swelling rate had elapsed. To remove excess saliva, the sample was then briefly placed on cellulose paper. Before applying the prepared specimen, a plate spacing of 30 mm was set. The sample was applied to the lower plate and the measurement gap of 1 mm was approached. The excess adhesive cream was removed via a spatula and discarded. To prevent drying out during measurement, a “liquid trap” was placed around the test setup.

### Calculation for Damping

To better assess how the physiological conditions of the oral cavity affect the mechanical properties of the adhesive cream, the increases or decreases in attenuation of one cream were evaluated as a percentage of the other cream. For this purpose, the calculated loss modulus values in the rheological tests were used and the percentage damping was calculated using Equation 1:
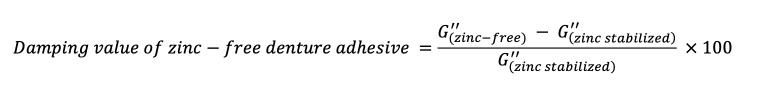


## RESULTS

As previously noted, experiments to determine the average time needed to attain a particular degree of swelling or swelling ratio for both the zinc-stabilized and zinc-free denture adhesive formulations were performed prior to rheological measurements. From the values determined there, it was possible to calculate the time for which a sample must be immersed in artificial saliva in order to attain a specific state of swelling.

The results are shown in Table 1 for the zinc-free denture adhesive formulation and in Table 2 for the zinc-stabilized denture adhesive formulation. The swell rate was initially rapid before tapering off as the adhesives became more saturated, with the variation in the degree of swelling becoming no longer significant in relation to the time period of immersion in artificial saliva. Some of the times to attain higher swelling percentages were not measured, as the swelling had already reached a saturation point by then and, beyond that point, the increase in swelling was found to be infinitesimal with respect to the time. This was especially the case for the zinc-stabilized formulation and was also observed for the zinc-free denture adhesive at pH 2 when measuring for a 120% swelling ratio (as omitted in Table 1).

**Table 1:** Time required in minutes to attain a specific degree of swelling (in percentage) for the zinc-free denture adhesive formulation

**23°C**	**Swelling (%)**
20	40	60	800	100	120
pH 2	time (min)	7.1	25.2	45.5	76.9	132.9	–
pH 7	time (min)	11.7	26.6	42.4	61.6	85.6	122.4
pH 10	time (min)	10.8	24.3	39.8	58.0	80.8	112.9


**Table 2:** Time required in minutes to attain a specific degree of swelling (in percentage) for the zinc-stabilized denture adhesive formulation

**23°C**	
20	40	60	80	100	120
pH 2	time (min)	31.2	65.6	119.1	–	–	–
pH 7	time (min)	13.4	31.8	57.0	97.8	154.8	–
pH 10	time (min)	15.3	33.7	56.4	86.3	132.1	–


**Fig 1a to f:**
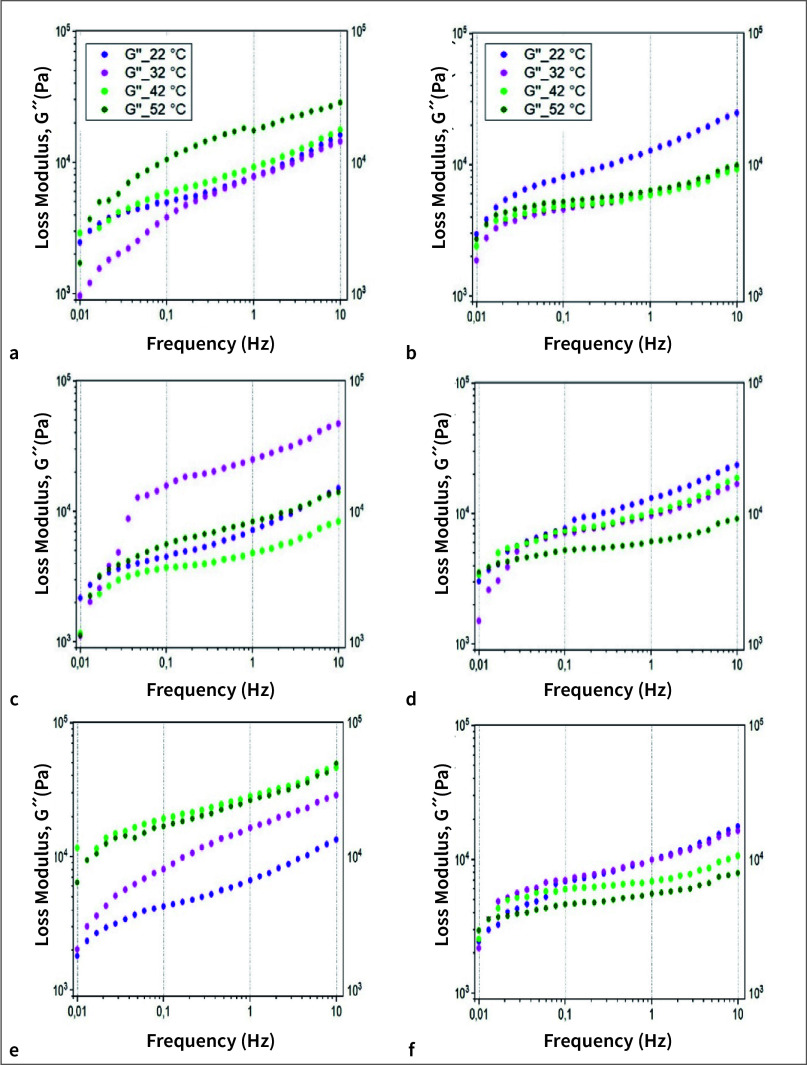
Comparison of the loss modulus for the two adhesive specimens at different temperatures in increments of 10°C. (a) At pH 2 and maximum swelling attained for zinc-stabilized adhesive; (b) at pH 2 and maximum swelling attained for zinc-free adhesive; (c) at pH 7 and maximum swelling attained for zinc-stabilized adhesive; (d) at pH 7 and maximum swelling attained for zinc-free adhesive; (e) at pH 10 and maximum swelling for zinc-stabilized adhesive; and (f) at pH 10 and maximum swelling for zinc-free adhesive.

Figure 1 shows the changes in loss modulus for the zinc-free and zinc-stabilized formulations in the given frequency range (0.01–10 Hz) at different temperatures. The results for the zinc-stabilized product are shown in Figures 1 (a), (c), and (e) and similarly for the zinc-free product shown in Figures 1 (b), (d), and (f). For visual clarity, only the values for 10°C temperature increments are shown. Figure 1 (a) shows the values of the loss moduli for the maximum swelling percentage for the zinc-stabilized denture adhesive at pH 2. Here it can be seen that the curves are less closely spaced in the lower frequency range. Moreover, G´´ is smallest here for the low temperatures and largest for 42°C. This trend changed at higher frequencies. The curves were again more compactly spaced, and the largest values were measured at 52°C. Similarly, Figure 1(b) describes the loss moduli for the zinc-free adhesive evaluated at the same temperature and pH values, as well as the corresponding maximum degree of swelling. Here, too, it can be seen that in the low frequency range the curves are very compact. With increasing frequency, the values for 32°C, 42°C, and 52°C are even nearly identical and the curves overlap. For a temperature of 22°C, the values for G´´ are largest. Figures 1(c) and (d) describe the results for the zinc-stabilized and zinc-free denture adhesives, respectively, at a neutral pH of 7 and temperature range akin to the above. Again, the results of the measurements are shown at maximum swelling percentage but here for a pH of 7. For the zinc-stabilized formulation at a temperature of 32°C, a very steep increase was seen in the smaller frequency range, as depicted in Figure 1(c). The remaining curves were approximately congruent with each other and were also relatively closer together. The smallest G´´ values occurred at 42°C. The curves for the zinc-free product showed hardly any temperature-related fluctuations when compared to the zinc-stabilized formulation, as shown in Figure 1(d). In the lower frequency range, it was also noted that the values of G´´ for high temperatures (52°C) were greatest. This changed with increasing frequency, and at higher frequencies the value of G´´ measured at 52°C was observed to be the smallest.

Furthermore, Figures 1(e) and (f) illustrate the results under the influence of an alkaline medium at pH 10 for the two adhesive specimens for the maximum swelling percentages. The plot in Figure 1(e) for the zinc-stabilized formulation demonstrated curves that were farther apart and showed maximum values at the temperatures of 42°C and 52°C. The lowest values were measured at a temperature of 22°C. The curves are approximately congruent. For the zinc-free formulation, temperature-dependent fluctuations are again evident from Figure 1(f), although relatively lower compared to the zinc-stabilized formulation. Here, in contrast to the zinc-stabilized formulation, the maximum values were measured at 22°C and the lowest values at 52°C. Analogous to the values for pH 7, once again the G´´ values measured at higher temperatures were greatest in the lower frequencies and the opposite being true for the G´´ values measured at lower temperatures. For all the graphs, the values for G´´ increased with increasing frequency and it was noticeable that there was usually a steeper increase for the range between 0.01 and 0.03 Hz as compared to the gradient for the higher frequency ranges. Extrapolating from the measured loss modulus values, the damping performance of the zinc-free denture adhesive formulation was compared with that of the zinc-stabilized formulation using Equation 1; the resulting data is tabulated in Table 3.

**Table 3:** Change in loss modulus as a metric of the relative damping of the zinc-free adhesive with respect to the zinc-stabilized adhesive

**Frequency**	Change in G´´ of zinc-free adhesive as a % of zinc-stabilized adhesive
Hz	22°C	32°C	42°C	52°C
0.01	40.1	35.1	86.6	–82.3
0.1	70.2	–55.1	19.4	–78.4
1	82.3	–61.2	50.2	–84.0
10	56.5	–64.3	40.9	–80.3


**Fig 2a to c:**
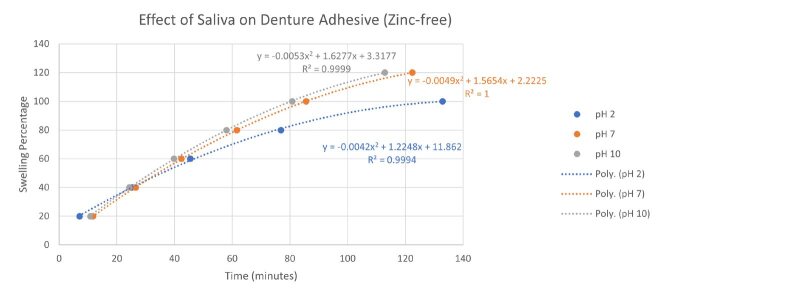

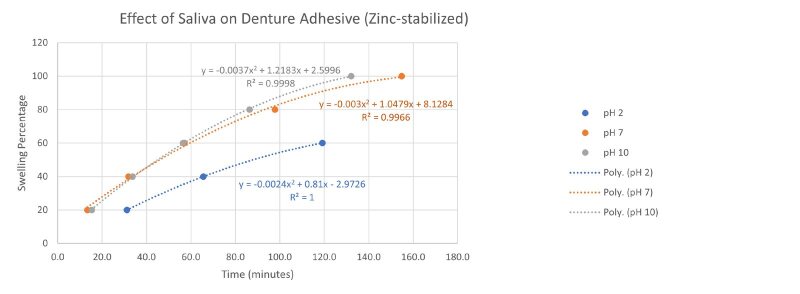

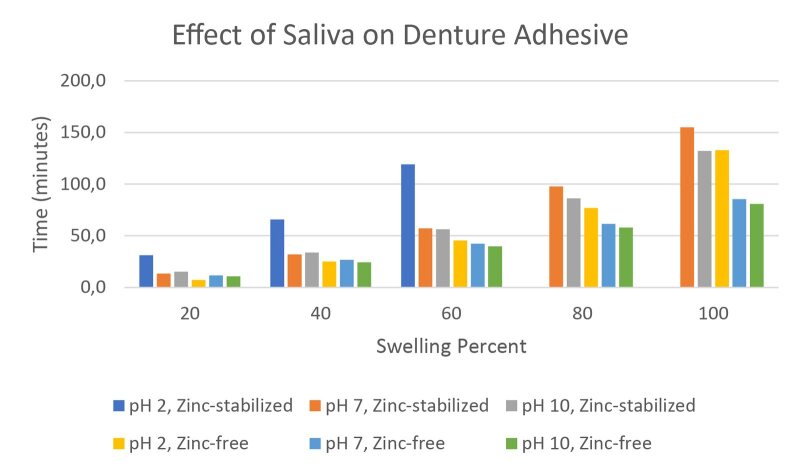
(a) Influence of pH and saliva on the swelling behavior of zinc-free denture adhesive; (b) Influence of pH and saliva on the swelling behavior of zinc-stabilized denture adhesive; and (c) Comparison of the influence of pH on the two denture adhesives.

**Fig 3:**
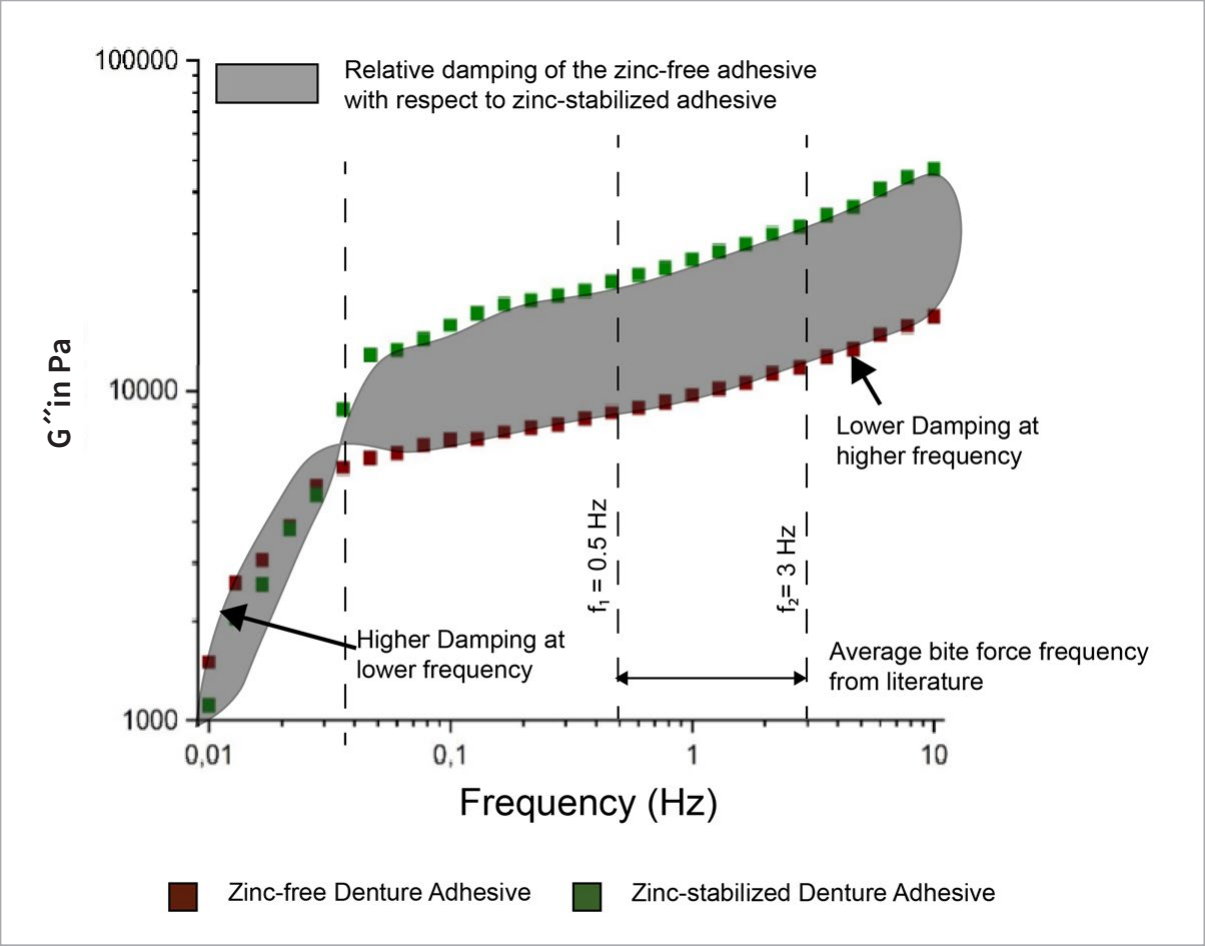
Comparison of the relative damping between the zinc-free and the zinc-stabilized denture adhesives in terms of the loss modulus values and with respect to increasing bite or chewing frequency.

These results indicated a significant variation of the damping behavior of the zinc-free formulation (calculated as a percentage with respect to the zinc-stabilized formulation). Additionally, the fluctuation clearly appeared to be dependent on both the temperature and the loading frequencies. A direct comparison of the influence of the pH on the swelling behavior of the zinc-free and zinc-stabilized adhesives based on the data from Table 1 and 2 is provided in Figure 2. From Figure 2(a) the influence of increasing pH can be visualized on the time required to attain a particular level of swelling for zinc-free denture adhesive. Similarly, Figure 2(b) illustrates the influence of pH on the zinc-stabilized denture adhesive. The two datasets are compared in Figure 2(c) which showed a low degree of influence of the pH on the adhesive’s swelling behavior in the case of zinc-free denture adhesives. The graphical interpretation of relative damping is exhibited in Figure 3, showing the loss modulus of the zinc-stabilized adhesive and the corresponding relative damping behavior of the zinc-free adhesive with respect to the zinc-stabilized adhesive in terms of increasing frequency.

## DISCUSSION

Based on the findings in this study presented in Figures 1, 2, and 3, the two formulations of zinc-stabilized and zinc-free denture adhesive show varying mechanical behavior in response to temperature, pH, and swelling of the adhesive under the influence of saliva. The relative damping values presented in Table 3 further strengthen the argument that the null hypothesis assumed at the beginning of the study can be rejected and the role played by the denture adhesive formulation including zinc in impacting the damping behavior is shown. Also, based on the results, we argue that the three input variables of temperature, pH, and swelling ratio have a strong influence on the mechanical behavior of the zinc-free and zinc-stabilized adhesive formulation.

Figures 1 (a)–(f) show the results of the rheological tests in the corresponding frequency range at different temperatures and pH values. In this study, we have focused only on the evaluation of the loss modulus, G´´ values, since it can be used to describe the damping properties of the denture adhesive formulation. Thus, conclusions can be made about the extent to which the forces transmitted through the denture to the oral mucosa during chewing are damped or not due to the presence of the respective denture adhesive in the oral cavity. By comparing these diagrams, it is possible to assess how the physiological conditions of the oral cavity influence the mechanical properties of the adhesive cream, and whether the two denture adhesive formulations investigated here are influenced to the same extent by each of them. First, the two formulations were considered individually. The influence of the pH value on the formulations can be visualized by comparing the gradients of the loss modulus values across the three selected pH values, which provides a good indication of the behavior of the adhesive in the acidic, neutral, and alkaline ranges. Other intermediate pH values could also be tested in the future to provide a more in-depth correlation and quantification of the influence of pH on the adhesive’s mechanical behavior.

This work provides data on the broad influence of pH at the extremes of the possible acidic and alkaline ranges of pH and compares the adhesive formulations over these extremes. The loss modulus plots from the zinc-stabilized and zinc-free denture adhesives must be compared both qualitatively and quantitatively. It can be seen that the measured G´´ values for the zinc-stabilized adhesive cream all lie within a range of 102 to 105 Pa based on the findings presented in Figure 1. Also, no significant deviations in the results are visible, irrespective of the pH value. This also applies to the zinc-free adhesive cream. For the zinc-free formulation it can also be seen that the range of values is different and includes G´´ values from 103 to 105 Pa. The influence of swelling cannot be assessed independently in this case, since only the maximum swelling rates were considered. However, the influence of temperature can be seen by looking at the individual graphs and comparing the curves. By doing so, one can see that the results for the zinc-stabilized formulation fluctuate more. This can be seen from the fact that the curves are less compactly spaced. At a pH of 7, the curves remain the most stable and lie furthest apart. The larger temperature-related fluctuations can be seen at pH 2 and 10. It is also noticeable that for pH 2 and pH 7, the values for G´´ increase with increasing temperature, ie, the damping properties increase. For the zinc-free adhesive cream, the values are quite stable across the changes in temperature, as all the curves are very close to each other. Additionally, the zinc-free formulation only demonstrates greater fluctuation at pH 2. Furthermore, with increasing temperature, the zinc-free G´´ values – and thus also the damping properties – decrease. Previous literature reports a similar damping influence of temperature and pH on the loss modulus of such adhesives.^18,32,47^ The results in Figures 2(a) and (b) illustrate that the pH and saliva in turn influence the degree of swelling attained by both the zinc-stabilized and the zinc-free denture adhesive formulations and further Figure 2(c) also highlights the relative deviation in the mechanical response due to the change in the formulation including the role played by zinc.

Nevertheless, individual consideration of the selected parameters and how they affect the respective adhesive creams is not in line with the true, combined physiological conditions of the oral cavity. *In vivo*, all such parameters act simultaneously on the denture and cream, and influence one another. Meanwhile, the steady state frequency-sweep-based shear rheological tests performed here were in the frequency range of 0.01 Hz to 10 Hz to best simulate the loads of a prosthesis within the oral cavity, as this is hypothesized to include the average biting or chewing frequency of human beings which is approximately in the range of 0.5 to 3 Hz.^11,12,19,20^ The extended testing range on both sides of this average is required to graphically interpret the mechanical behavior of the adhesive formulation. As such, the measurements were performed over three decades of measurement between 0.01 Hz and 10 Hz, with each decade including 10 logarithmically spaced measurement points. In this way, all load states of a prosthesis are represented. Lower frequencies can hypothetically represent the unloaded state, while certain increased frequencies occur during chewing. This depends on, among other things, the progression of the mastication process, the volume of the food bolus, and the size of the individual food particles. Po et al^35^ determined a variation in chewing frequency of 0.9 to 2.15 Hz among their subjects, with a mean value of 1.85 Hz. The range of 0.01 to 10 Hz used in this study extends this and is intended to represent the following loading states of prosthesis: 10 Hz fast chewing, 1 Hz slow chewing, 0.01 Hz resting.

Previously, the literature has generally not focused on the relative damping performance of denture adhesives and the correspondingly reduced pain sensation for denture wearers. Most reports predominantly concentrate on the enhancement of denture retention behavior from using denture adhesives.^24,39,51^ From the results presented here in Table 3, however, we can compare the relative damping of the two tested adhesives. The zinc-free denture adhesive showed a higher loss modulus for all the pH measurements at lower temperatures (22°C) compared to the zinc-stabilized denture adhesive. Thus, the zinc-free adhesive was found to have a higher resistance to deformation as well as a higher viscosity. In other words, the zinc-free adhesive potentially provides much higher damping to the oral cavity under the influence of an applied bite or chewing force. As a result, this could lower the sensation of pain in denture wearers at lower temperatures in the oral cavity, such as when consuming cold foods. Further, the relative damping was noted to increase with increasing bite frequency. However, the pattern in the relative damping behavior was found to be reversed at higher temperatures (52°C), where the loss modulus values of the zinc-free adhesive were much lower compared to the zinc-stabilized adhesive. This implies that at higher temperatures, the zinc-free adhesive has a lower resistance to distortion and hence lower damping of the applied bite force compared to the zinc-stabilized adhesive. The zinc-free adhesive once again exhibited a higher damping or resistance to distortion at lower bite or chewing frequencies, as observed in Figure 3. Thus, the zinc-free adhesive data in this study indicates that this formulation type may lead to lower contact pressures on the oral mucosa and, therefore, a lower perception of pain. The zinc-free adhesive’s behavior reversed at frequencies above 0.07 Hz and tended toward lower resistance to distortion compared to the zinc-stabilized adhesive under the physiological factors considered in this study. The zinc-stabilized adhesive, based on the investigations in this study, exhibited a higher damping at higher bite or chewing frequencies. The damping factor has been shown in previous studies to lower the stresses on the underlying soft tissues and, as a consequence, result in lower levels of pain in denture wearers.^1,6,45,46,50^

## CONCLUSIONS

Based on the findings of this study, the following conclusions were drawn:

The tested zinc-free denture adhesive formulation provided higher damping at lower frequencies of applied force and, as a consequence, it is argued to potentially lower the pain experienced by denture wearers.
However, this trend was seen to reverse at higher frequencies, and this has to be investigated in further detail.
The varying impact of physiological parameters evaluated in this study – temperature, pH, and degree of swelling under the influence of saliva on both the zinc-free and zinc-stabilized denture adhesive formulations – indicate the influence of zinc and other constituents on their respective mechanical behavior.

As such, our results can inform the consideration of denture adhesive cream formulations, as well as potential avenues of altering their mechanical behavior to improve their performance.

## REFERENCES
